# A Novel Latin Hypercube Algorithm via Translational Propagation

**DOI:** 10.1155/2014/163949

**Published:** 2014-09-02

**Authors:** Guang Pan, Pengcheng Ye, Peng Wang

**Affiliations:** School of Marine Science and Technology, Northwestern Polytechnical University, Xi'an 710072, China

## Abstract

Metamodels have been widely used in engineering design to facilitate analysis and optimization of complex systems that involve computationally expensive simulation programs. The accuracy of metamodels is directly related to the experimental designs used. Optimal Latin hypercube designs are frequently used and have been shown to have good space-filling and projective properties. However, the high cost in constructing them limits their use. In this paper, a methodology for creating novel Latin hypercube designs via translational propagation and successive local enumeration algorithm (TPSLE) is developed without using formal optimization. TPSLE algorithm is based on the inspiration that a near optimal Latin Hypercube design can be constructed by a simple initial block with a few points generated by algorithm SLE as a building block. In fact, TPSLE algorithm offers a balanced trade-off between the efficiency and sampling performance. The proposed algorithm is compared to two existing algorithms and is found to be much more efficient in terms of the computation time and has acceptable space-filling and projective properties.

## 1. Introduction

In engineering, manufacturing companies strive to produce better and cheaper products more quickly. However, engineering systems are fairly large and complicated nowadays. In addition, design requirements are rigorous and stringent for such systems, especially multidiscipline design optimization systems such as aerospace. These engineering analysis and design problems usually involve expensive computer simulations. For example, it is reported that it takes Ford Motor Company about 36–160 h to run one crash simulation [1], which is unacceptable in practice. Although the capacity of computer keeps increasing, the complexity of analysis software, for example, finite element analysis (FEA) and computational fluid dynamics (CFD), seems to keep pace with computing advances [2]. To alleviate the computational burden, metamodels, which are often called surrogate models or response surfaces, are widely used for optimization and design analysis by creating approximate models to replace the expensive computer simulations. Because the accuracy of metamodels directly depends on the samples of computer simulations, it is important to obtain efficient designs of computer experiments.

In recent decades, various sampling designs have been developed for computer experiments. The classical experiment designs containing alphabetical optimal design [3], factorial or fractional factorial design [4], central composite design (CCD) [5], and so forth, were widely used earlier. However, they do not have good performance of both space-filling and projective properties. As is recognized by many researchers, designs for computer experiments should at least satisfy the following two criteria (see [6–10]). Firstly, the design should be space-filling in some sense. When no details on the functional behavior of the response parameters are available, it is necessary to be capable of obtaining information from the entire design space. Therefore, design points should be “evenly spread” over the entire region. Secondly, the design should be noncollapsing. When one of the design variables has almost no effect on the function value, two design points that differ only in this variable will “collapse”; that is, they can be considered as the same point that is evaluated twice. As evaluation of the deterministic black-box function is often time-consuming, this is not a desirable situation. Therefore, two design points should not share any coordinate values when it is not known a priori which dimensions are important. Furthermore, we would like the projections of the points onto the axes to be separated as much as possible. Based on these two properties, a space-filling Latin hypercube design termed LHD in this paper is an appropriate and popular choice.

Latin hypercube designs (LHD) play an important role in computer experiments. The Latin hypercube structure allows one to achieve both the space-filling requirement and the noncollapsing condition. Each column of an *n*-dimensional LHD of *m* points is a random permutation of {1,2,…, *m*}. By scaling, we can use LHD for any rectangular design space. An LHD has good projective properties on any single dimension but bad space-filling properties when it is randomly selected. To further obtain the good space-filling property, the optimal LHD is widely studied. Koehler and Owen [11] showed that the projection of the optimal LHD onto a subset of variables retains good spatial properties. Morris and Mitchell [7] employed the simulated annealing (SA) algorithm for constructing optimal LHD. Ye et al. [12] made a research on the columnwise-pairwise (CP) algorithm for constructing optimal symmetrical LHD. Jin et al. [13] introduced the enhanced stochastic evolutionary (ESE) algorithm for finding various space-filling designs, including approximate maximin LHD. Bates [14] described a method for generating optimal LHD using PermGA by minimizing the potential energy *U*. Liefvendahl and Stochi [15] compared the efficiency of CP and genetic algorithm (GA) for the optimization of LHD. Grosso et al. [16] adopted iterated local search (ILS) for improving the objective function *ϕ*_*p*_ to obtain maximin LHD problem. Jourdan and Franco [17] presented an optimal LHD using the Kullback-Leibler criterion.

Although aforementioned methods provide effective ways to produce samples with good space-filling and projective properties, they are computationally inefficient for problems with large dimensions and sample sizes. For example, Ye et al. [12] reported that generating an optimal 25 × 4 LHD using CP could take several hours on a Sun SPARC 20 workstation. The search for a larger design would take even longer and may be computationally prohibitive. Thus, search processes often stopped before finding a good design. This situation motivated us to look for alternatives that require less computing time. In recent years, some methods without expensive optimization procedures were investigated. Van Dam et al. [18] presented some general formulas to obtain maximin LHD, which is just used for two-dimensional problems and limited by the number of sampling points. Viana et al. [19] presented a new method to obtain near optimal LHD without going through the expensive optimization process, whereas the projective property of the sampling points is not satisfying except for some special problem sizes. Zhu et al. [20] presented a novel algorithm of maximin Latin hypercube design using successive local enumeration.

In this paper, we propose a method that is able to quickly construct a good design of experiments given a limited computational resource. There are two major algorithms involved. One is translational propagation algorithm (TP) [19], which requires minimal computational effort and does not use formal optimization. It can solve the optimization problem in an approximate sense, that is, to obtain a good Latin hypercube quickly, rather than finding the best possible solution. The other is successive local enumeration algorithm (SLE) [20]. It can maximize the minimal distance which is the minimum of all the distances between the point to be generated and the existing points. The sampling points produced by SLE are evenly distributed in the design space and projective points in lower dimensions are almost uniform [20]. The algorithm proposed in this paper is a combination of TP algorithm and SLE algorithm which is termed TPSLE. In fact, it is a compromise between computing efficiency and sampling performance, that is, space-filling properties and projective properties. TPSLE algorithm is based on the inspiration that a near optimal Latin hypercube design can be constructed by a simple initial block as a building block with a few points generated by algorithm SLE. Testing results compared with the MATLAB function LHSDESIGN and SLE indicate that this method is effective to generate sampling points with good space-filling and projective properties. In addition, the sampling efficiency of TPSLE is the highest through comparison with function LHSDESIGN and SLE. In this paper, MATLAB function LHSDESIGN is termed LHSD.

The remainder of the paper is organized as follows. The proposed TPSLE algorithm for obtaining Latin hypercube designs is described in Section 2, and then testing results compared with LHSD and SLE are represented to show its acceptable sampling performance and high efficiency in Section 3.  Section 4 provides the further comparative study to show the advantages of the proposed TPSLE algorithm on improving the metamodels accuracy and solving mechanical design optimization problem. Eventually, conclusions are drawn in Section 5, where the shortcomings of TPSLE and future works are also pointed out.

## 2. Description of TPSLE Algorithm

In order to illuminate the algorithm in detail, the basic procedure of TPSLE is introduced first, followed by the application of the novel algorithm for a two-dimensional problem. Then a method of generating experimental designs of any size is proposed. Certainly the summary of TPSLE algorithm will be given at last.

### 2.1. Basic Process of TPSLE Algorithm

The proposed algorithm is based on the inspiration of constructing the *n*-dimensional Latin hypercube design from a fairly small optimal *n*-dimensional Latin hypercube design used as an initial block via translational propagation [20]. In order to strengthen understanding, a simple example of a size 16 × 2 (i.e., sixteen sampling points in two dimensions) Latin hypercube design is used to elaborate the methodology below.

Assuming to construct a Latin hypercube design of *m*_*p*_ points and *n*_*p*_ dimensions from an initial block design of *m*_*b*_ points and *n*_*b*_ dimensions, each dimension is partitioned into the same number of divisions as 2. So the design space is divided into a total of *b* blocks such that

(1) $$\begin{array}{c} b=2^{n_{p}}. \end{array}$$

Meanwhile, the number of points *m*_*b*_ of block design is defined as

(2) $$\begin{array}{c} m_{b}=\frac{m_{p}}{b}=\frac{m_{p}}{2^{n_{p}}}, \end{array}$$

where dimensions of block design *n*_*b*_ should be equal to the dimensions of Latin hypercube design *n*_*p*_; that is, *n*_*b*_ = *n*_*p*_.

In the example of the 16 × 2 Latin hypercube design (i.e., *m*_*p*_ = 16 and *n*_*p*_ = 2), one obtains *n*_*b*_ = 2, *b* = 4, and *m*_*p*_ = 4 from (1) and (2).

**Figure 1:**
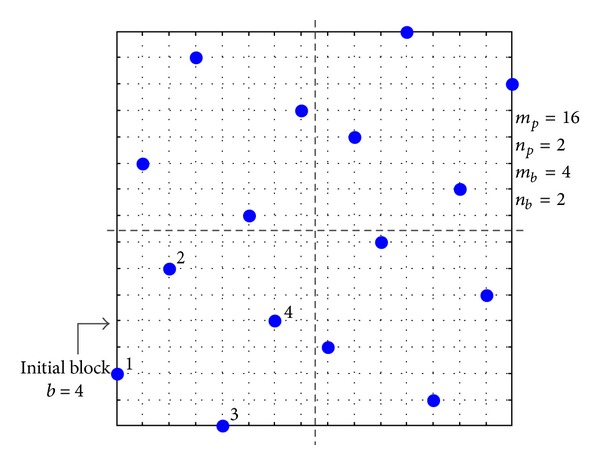
16 × 2 Latin hypercube mesh divided into blocks (2 divisions in each dimension results in 4 blocks). The left-lower block is the initial block to be first picked in the algorithm.

**Figure 2:**
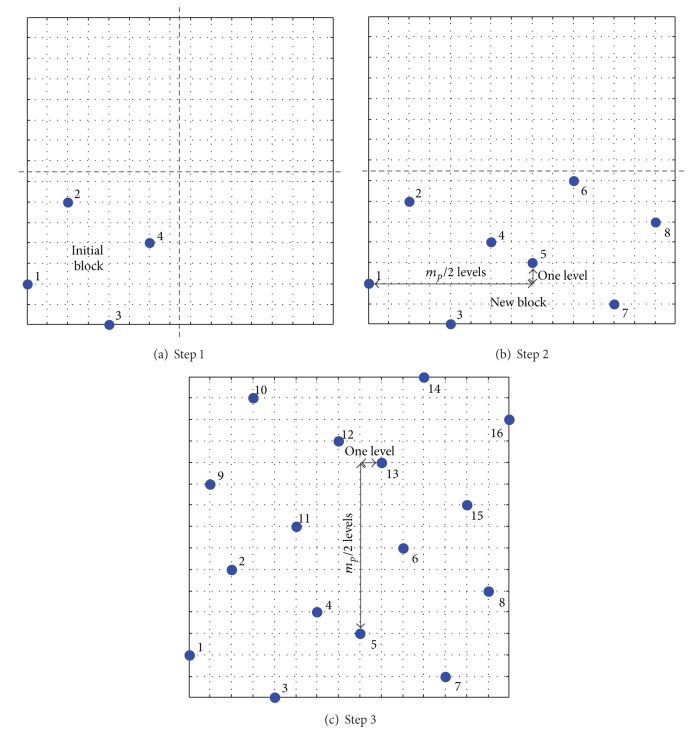
Process of creating the 16 × 2 Latin hypercube design. (a) illustrates the initial block. (b) shows the translation of the initial block in the horizontal direction with *m*_*p*_/2 levels which is accompanied by a one-level vertical displacement to preserve Latin hypercube properties and represents the newly created block that will be translated in the vertical direction. (c) shows the translation in the vertical direction which is accompanied by horizontal displacement of one level.

Next, points of initial block are generated by the optimal Latin hypercube design SLE which will be introduced in the next section. Then the entire design space will be filled with the initial block via translational propagation algorithm.  Figure 1 shows the division of the design space for the 16 × 2 Latin hypercube design.  Figure 2 illustrates the process step by step. First, the initial block is properly filled with points determined by SLE algorithm as shown in Figure 2(a). Next, the initial block is shifted by *m*_*p*_/2 levels in one of the dimensions. Every time that the old block is shifted, a new block is added to the experimental design to produce a new block (twice as points of old block).  Figure 2(b) shows the shift of the initial block (chosen to be in the horizontal direction). To preserve the noncollapsing property of Latin hypercube, that is, only a single point per level, there also has to be a one-level shift in the vertical direction which is shown in Figure 2(b). In the general case, a displacement vector is built for each accounting for the shifting in the dimension of interest (horizontal direction in the example above) as well as a shift in all other dimensions to preserve the Latin hypercube properties (vertical direction in our example). In the next step, the current set of points (newly filled division) is used as a new block and the procedure of shifting the block is repeated in the next dimension.  Figure 2(c) illustrates the shifting procedure in the vertical direction.

The greatest advantage of this approach is that there are no calculations to perform once initial block is completed. All operations can be viewed as a simple translation of the block designs in the *n*_*p*_-dimensional hypercube. Although efficient for generating sampling designs, the algorithm proposed now fails to provide flexibility to obtain any sample size in the final Latin hypercube design. Equations (1) and (2) must hold, that is, become responsible for the limitation of algorithm. The strategy to overcome this limitation and generate sample designs with arbitrary size is described in Section 2.3.

### 2.2. Novel Optimal Algorithm of Latin Hypercube Design SLE

**Figure 3:**
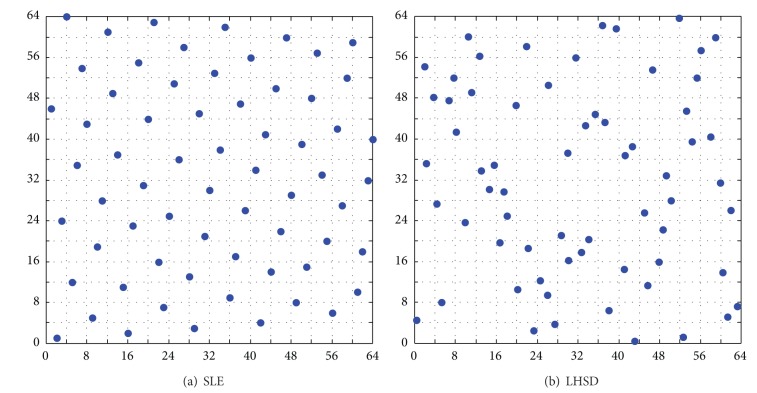
Sampling comparison with MATLAB function LHSD (*m*_*b*_ = 64, *n*_*b*_ = 2).

In this section a novel algorithm of maximin LHD using SLE is introduced briefly, referred to in [19]. Unlike the existing LHD methods which employed the global objective functions, the sequential local objective function is to maximize the minimal distance which is the minimum of all the distances between the point to be generated and the existing points already generated by SLE. The points produced by this method are evenly distributed in the design space and projective points in lower dimensions are almost uniform. Based on SLE algorithm, the sampling points in a two-dimensional plane are shown in Figure 3(a). The projective points to each coordinate axis are uniform. Comparison with LHSD function provided by MATLAB using the default set is shown in Figure 3(b). From the comparative plots, sampling points generated by using SLE algorithm have better space-filling and projective property.

Similarly, assuming to generate an initial Latin hypercube design of *m*_*b*_ sampling points and *n*_*b*_ dimensions by SLE algorithm. This problem of finding a set of sampling points in *n*_*b*_-dimensional space can be described as positioning *m*_*b*_ points in a *m*_*b*_^*n*_*b*_^ unit hypercube, each point in which has *n*_*b*_ coordinates values, (*x*_*i*1_, *x*_*i*2_,…, *x*_*in*_*b*__) ∈ {1,2,…,*m*_*b*_}^*n*_*b*_^, (*i* = 1,2,…, *m*_*b*_), so that all the *m*_*b*_ points possess good performance, that is, space-filling and projective properties. According to the SLE algorithm, the design space will be divided into the *m*_*b*_^*n*_*b*_^ unit hypercube. The sampling points should be determined cell by cell (when *n*_*b*_ = 2, a cell is equal to a column), and for each cell only one point can be designated. A cell can be considered as a (*m*_*b*_−1)^*n*_*b*_^ unit hypercube, which owns (*m*_*b*_−1)^*n*_*b*_^ hyperboxes (when *n*_*b*_ = 2, a hyperbox is equal to a square, and when *n*_*b*_ = 3, a hyperbox is equal to a cube).

When using SLE algorithm to construct an initial block for TPSLE algorithm, it is noticed that the variable (*x*_*i*1_, *x*_*i*2_,…, *x*_*in*_*b*__) ∈ {1+(0,2^*n*_*b*_−1^,2∗2^*n*_*b*_−1^,…,(*m*_*b*_−1)∗2^*n*_*b*_−1^)}^*n*_*b*_^, (*i* = 1,2,…, *m*_*b*_), which is different from the SLE algorithm in the literature [19]. Figure 2(a) shows that the interval of points in initial block is two-level which is equal to 2^*n*_*b*_−1^ = 2.

### 2.3. Constructing Designs of Experiment with Arbitrary Size

To generate an improved Latin hypercube design proposed in this paper with any number of points, the first step is to generate a TPSLE that has more points than the required. The experimental design will be completed without resizing the size of TPSLE if the design of *m*_*p*_ points and *n*_*p*_ dimensions is proper; that is, *m*_*b*_ calculated by (2) is an integer. Otherwise, an experimental design which is larger than the required will be created through rounding *m*_*b*_ up. And then a resizing process will be used to reduce the number of points to the desired one. The points are removed one by one from the initially created TPSLE by discarding the points that are the furthest from the center of the hypercube and reallocating remaining points to fill the whole design (preserving the Latin hypercube properties). In the proposed algorithm removing the points furthest from the center does not reduce the area of exploration. After removing the points, the final design is rescaled to cover the whole design space. The detailed process of resizing algorithm refers to the literature [20]. In the next paragraph, an experimental design of Latin hypercube with a size 13 × 2 will be illustrated step by step.

**Figure 4:**
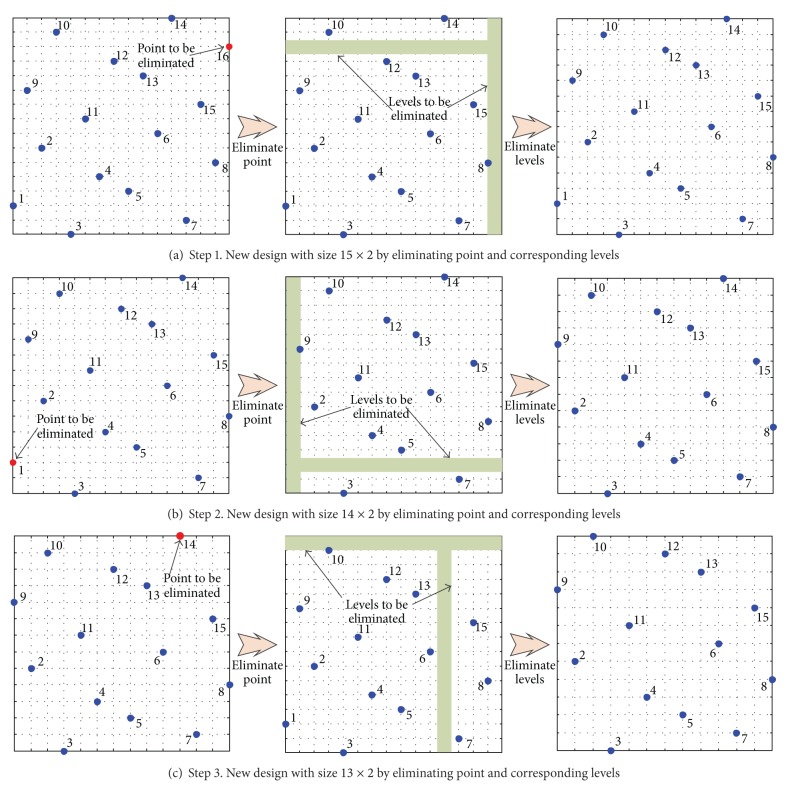
Process of resizing to create a 13 × 2 size design.

To construct a 13 × 2 size Latin hypercube (i.e., *m*_*p*_ = 13 and *n*_*p*_ = 2), the corresponding initial block should be created first. From (1) and (2), one can obtain *m*_*b*_ = *m*_*p*_/2^*n*_*p*_^ = 3.25 which is not an integer. Rounding *m*_*b*_ = 3.25 up to *m*_*b*_ = 4, then the size of larger design that can be constructed is 16 × 2, as illustrated in Figure 1. The resizing process begins with first calculating the distance between each of the 16 points and the center of the design space. To create a 13 × 2 size design out of a 16 × 2 one, three points furthest from the center have to be eliminated. In practice, this means the points of the original TPSLE have to be ranked according to the distance between the original points and the center of the design space. Three points which are further from the center are eliminated gradually. When two points are equally far from the center, it is not important which of the points will be removed first due to symmetry. In general, the point which is further from the origin will be removed. Certainly, the point which is nearer to the origin can also be chosen to be removed. The red point marked with 16 is first eliminated rather than point 1, as illustrated in Figure 4. However, removing point merely may break the Latin hypercube property that only a single point is found at any of the levels. So once a point is removed, the levels occupied by its projection along each of the dimensions have to be eliminated. Figure 4 illustrates the resizing process step by step. The number of points in the design progressively shrinks, but the final design still represents samples over the same design space.  Figure 4(a) shows that in the 16 × 2 design the red point which is farther from the center and its corresponding levels marked with green shadow are eliminated. When eliminating levels, the remaining points are used to occupy the empty level that is in between the points remarked with 10, 12 in Figure 4(a). In Figure 4(a), two points marked with 10, 14 on the top would move downward to occupy the empty level. Next, all points are scaled to cover the original design space. After each step, a new Latin hypercube design is obtained with one point less. The process continues until the 13 × 2 design is achieved.  Figure 4(b) and Figure 4(c) display the same process of eliminating points marked with 1, 14 and their corresponding levels. Removing points/levels part reduces the number of points of the experimental designs to obtain sample designs with arbitrary size, while preserving the Latin hypercube properties. The corresponding dimensions will not be eliminated after one certain point is chosen to be eliminated. On the other hand, it makes the experimental design fit in the original design space again.

### 2.4. Summary of the TPSLE Algorithm

**Figure 5:**
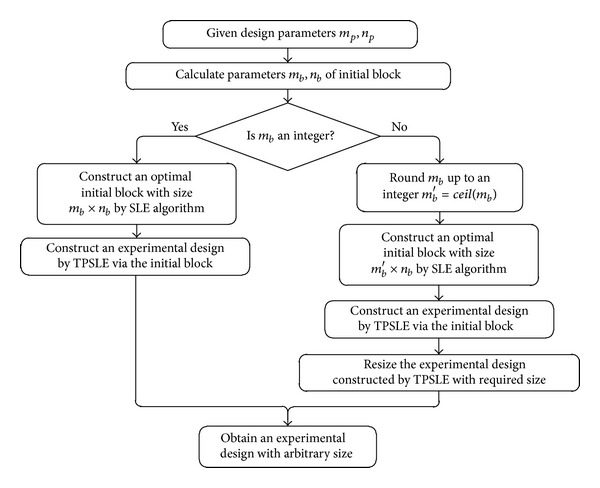
Flowchart of proposed algorithm TPSLE.

The proposed algorithm is inspired by the trade-off between performance and efficiency of experimental sampling design. In practice, good Latin hypercube designs are expected to be obtained efficiently because the consuming time is limited. This is particularly critical for large number of points in high dimensions. TPSLE generated from a fairly small optimal Latin hypercube design used as an initial block via translational propagation algorithm is a superior design relatively.  Figure 5 illustrates the TPSLE algorithm. The given design parameters include the number of points of the required Latin hypercube design *m*_*p*_ and the number of variables *n*_*p*_. The first step is to calculate the design variables *m*_*b*_, *n*_*b*_ and number of blocks *b* from (1) and (2). Then checking whether *m*_*b*_ is an integer. If *m*_*b*_ is an integer, the initial block will be constructed with size *m*_*b*_ × *n*_*b*_ by SLE algorithm. Afterwards, the required experimental design is constructed by TPSLE algorithm via translational propagation. However, while *m*_*b*_is not an integer, the initial block cannot be constructed immediately. It is advised to round *m*_*b*_ up; *ceil*() in Figure 5 represents the rounding up. In this algorithm, parameter *m*_*b*_ controls the number of sampling points, that is, *m*_*p*_ = *m*_*b*_ · 2^*n*_*p*_^. Thus, a larger Latin hypercube design is obtained using the initial block via translational propagation algorithm. Next, the Latin hypercube design is resized to the required one. So far, the process of constructing a Latin hypercube design with the arbitrary size is completed and the required experimental design is achieved.

**Figure 6:**
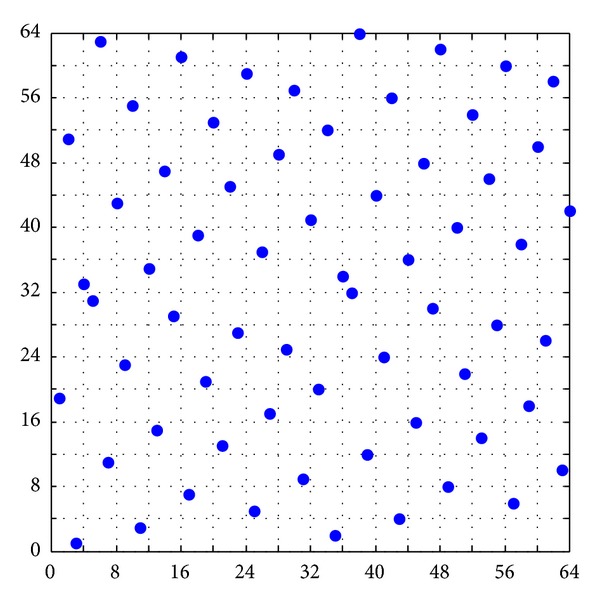
A Latin hypercube design with size 64 × 2 by TPSLE.

Based on the abovementioned TPSLE algorithm, the sampling points illustrated in Figure 6 in two-dimensional space with size 64 × 2 are generated, compared with the sampling points produced by SLE and LHSD which are shown in Figure 3. From the comparative plots, the space-filling and projective properties of sampling design generated by using the TPSLE algorithm are better than LHSD and coincident with SLE roughly, but the efficiency of TPSLE algorithm is farther superior to SLE algorithm which will be discussed in the next section.

## 3. Results and Discussion

The sampling points generated by the TPSLE algorithm meet the two desired features, namely, space-filling and projective properties. The distributions of the produced sampling points are even in the design space and the projective points in lower dimensions are almost uniform, especially for projecting to each coordinate axis. According to the sampling process of the TPSLE algorithm, the initial block constructed by SLE is used to generate the sampling points via translational propagation, which are quite different from the existing LHD sampling methods. In TPSLE, there are no global objective functions, such as *ϕ*_*p*_, potential energy *U* to optimize and thus no expensive optimization algorithm such as genetic algorithm and simulated annealing would be employed, so the efficiency of the algorithm TPSLE is superior to the sampling methods containing optimization algorithms. In this section, the performance and efficiency of algorithm TPSLE are both tested compared with two existing Latin hypercube algorithms.

### 3.1. Test Criteria

In recent years, some optimal criteria are employed widely to achieve a good performance in design of computer experiments. The optimal designs constructed by these optimal criteria have been shown to have a good performance. In other words, these optimal criteria can be used as test criteria to test whether the experimental designs have good performance. Four widely used test criteria are considered in this work.

#### 3.1.1. Maximin Distance Criterion *d*_min⁡_

Maximin distance criterion is proposed by Johnson et al. [6]. As the term suggests, the objective of the criterion is maximizing the minimum intersite distance *d*_min⁡_:

(3) $$\begin{array}{c} d_{\min}=\underset{1\leq i,j\leq m,\;i\neq j}{\min}d\left(x_{i},x_{j}\right), \end{array}$$

where *m* is the number of points and *d*(*x*_*i*_, *x*_*j*_) is the distance between two arbitrary points:

(4) $$\begin{array}{c} d\left(x_{i},x_{j}\right)=d_{ij}={\left[\overset{n}{\underset{k=1}{\sum }}{\left|x_{ik}-x_{jk}\right|}^{t}\right]}^{1/t},\;t=1\;\;\text{or}\;\;2, \end{array}$$

where *n* is the number of variables. In this paper, *t* = 2 is considered. The parameters *d*_*ij*_, *m*, and *n* are the same as those used for test criteria below.

#### 3.1.2. Centered *L*_2_ Discrepancy Criterion *CL*_2_

Centered *L*_2_ discrepancy criterion is one of *L*_*p*_ discrepancy criteria which is a measure of the difference between the uniform cumulative distribution function and the empirical cumulative distribution function of an experimental design. Namely, the *L*_2_ discrepancy is a measure of nonuniformity of a design that is used most widely. Hickernell [21] proposed an interesting formula of *L*_2_ discrepancy termed as centered *L*_2_ discrepancy *CL*_2_ expressed as follows:

(5) CL2(X) =(1312)n−2m∑i=1m ∏k=1n(1+12|xik−0.5|−12|xik−0.5|2)  +1m2∑i=1m ∑j=1m ∏k=1n(1+12|xik−0.5|+12|xjk−0.5|−12|xik−xjk|).

#### 3.1.3. *ϕ*_*p*_ Criterion

In 1995, Morris and Mitchell [7] proposed an intuitively appealing extension of the maximin distance criterion:

(6) $$\begin{array}{c} \phi_{p}={\left[\overset{s}{\underset{i=1}{\sum }}J_{i}{d_{i}}^{-p}\right]}^{1/p}, \end{array}$$

where *d*_*i*_ are distinct distance values with *d*_1_ < *d*_2_ < ⋯<*d*_*s*_, *J*_*i*_ is the number of pairs of sites in the design separated by *d*_*i*_, *p* is a positive integer, and *s* is the number of distinct distance values. Jin et al. [13] provided a new equation to efficiently evaluate the value of *ϕ*_*p*_ which is expressed by

(7) $$\begin{array}{c} \phi_{p}={\left[\underset{1\leq i,j\leq m,\;i\neq j}{\sum }{d_{ij}}^{-p}\right]}^{1/p}, \end{array}$$

where *d*_*ij*_ can be obtained by (4) and *p* = 50 are advised by the literature [13].

#### 3.1.4. Potential Energy Criterion *U*

In optimal LHD algorithms, the Audze-Eglais objective function [17], namely, the potential energy criterion *U*, is usually used as a criterion for checking whether sampling points have good performance. It is inspired by the following physical analogy: a system will reach equilibrium when the potential energy of the repulsive forces between the masses is at a minimum. The potential energy criterion *U* is inversely proportional to the distance squared between the points formulated as follows:

(8) $$\begin{array}{c} U=\overset{m-1}{\underset{i=1}{\sum }}\;\overset{m}{\underset{j=i+1}{\sum }}d_{ij}^{-2}. \end{array}$$

### 3.2. Performance of TPSLE Algorithm

To illustrate the performance, that is, space-filling and projective properties of the sampling points, four aforementioned criteria are employed, namely, *d*_min⁡_, *CL*_2_, *ϕ*_*p*_, and potential energy *U*, to compare with other existing LHD methods. In this work, LHSD function in MATLAB and SLE algorithm [19] are used to make a comparison with TPSLE algorithm.

Various sampling designs are generated by three different Latin hypercube design methods including TPSLE, LHSD, and SLE. In order to reduce the randomness of sampling designs, sampling points are generated for 50 times through the TPSLE and SLE algorithm. Meanwhile, 500 times are for LHSD algorithm with the default set in MATLAB. It is noticed that sampling points are generated for 10 times as *n* ≥ 10. And the best, worst, and mean values of the different criteria are calculated. It is noticed that the sampling designs are scaled to 0~1. Afterwards, testing and comparison results based on four test criteria which are minimal distance *d*_min⁡_, centered discrepancy *CL*_2_, *ϕ*_*p*_, and potential energy *U* are shown in Tables 1 and 2. The larger the values of *d*_min⁡_ and the smaller the values of *CL*_2_, *ϕ*_*p*_, and *U* which are marked with bold and italic in Tables 1–3, the better the sampling design.

According to the comparison study with SLE algorithm and LHSD function with various number of points in two-dimension in Table 1, the most mean values of *CL*_2_, *ϕ*_*p*_, and *U* of the sampling designs produced by TPSLE algorithm are smaller than LHSD function, and the mean values of *d*_min⁡_ of sampling designs produced by TPSLE algorithm are all larger than LHSD function, which demonstrate that sampling designs using TPSLE algorithm have better performance compared with LHSD function. Furthermore, part of worst values *d*_min⁡_, *CL*_2_, *ϕ*_*p*_, and *U* of sampling designs produced by TPSLE algorithm are better than mean values of those produced by LHSD function especially for the criteria *d*_min⁡_ and *U*. In order to show the good performance of sampling designs produced by TPSLE algorithm comprehensively, comparisons are made with sampling designs produced by SLE algorithm which is a time-consuming algorithm. From the results shown in Table 1, sampling designs produced by TPSLE algorithm are compared to SLE algorithm in terms of performance. It is attractive that the sampling design with size 32 × 2 generated by TPSLE algorithm is better than the other two methods in terms of test criteria *d*_min⁡_, *ϕ*_*p*_, and *U*.

**Figure 7:**
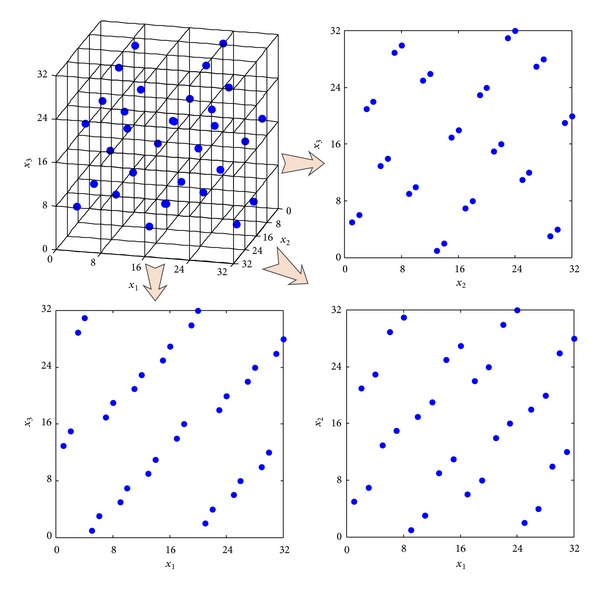
3D space-filling and corresponding 2D projective points generated by TPSLE.

**Figure 8:**
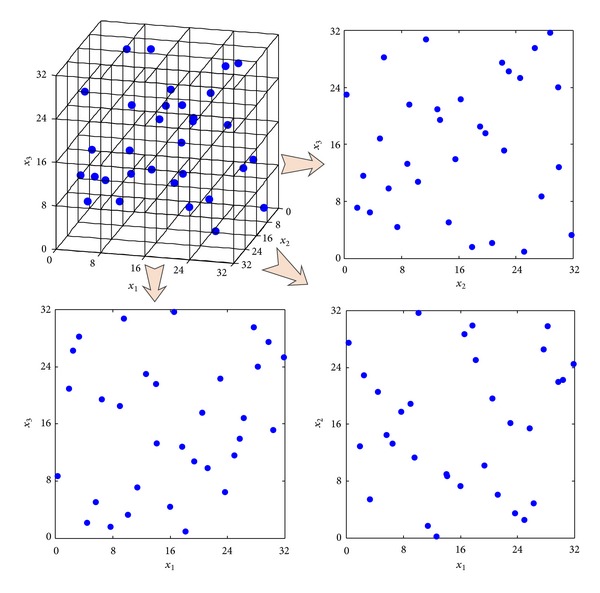
3D space-filling and corresponding 2D projective points generated by LHSD.

**Figure 9:**
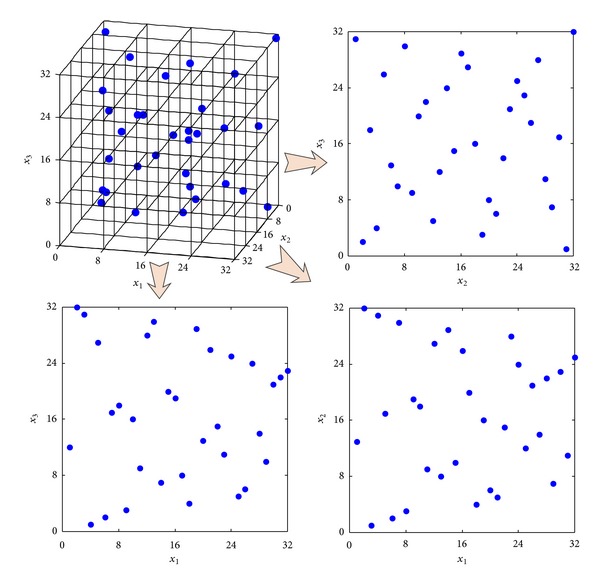
3D space-filling and corresponding 2D projective points generated by SLE.

Similarly, the results of sampling designs in three-dimension from Table 2 demonstrate the same conclusion as aforementioned. For observing the performance of sampling design produced by TPSLE algorithm intuitionally, the 3D space-filling and corresponding 2D projective points generated based on three sampling methods separately are shown in Figures 7, 8, and 9.

For the sake of reflecting good performance of TPSLE further, the test criteria *d*_min⁡_, *ϕ*_*p*_, and *U* of TPSLE are studied to compare with LHSD in high dimension, as shown in Table 3. As it is shown in Table 3, the minimum distances between any two points *d*_min⁡_ of sampling designs in high dimension generated by TPSLE are all larger than LHSD. The other criterion *ϕ*_*p*_ is smaller compared with LHSD. However, the potential energy *U* of sampling designs generated by LHSD is smaller in some cases. It indicates that different optimal sampling designs may be obtained based on different optimal criteria. According to the comparison in Table 3, the performance of sampling designs generated by TPSLE in most cases is better than LHSD in high dimension.

In a word, we can conclude that better space-filling and projective properties can be obtained by TPSLE through comparison with LHSD and SLE under different criteria of *d*_min⁡_, *CL*_2_, *ϕ*_*p*_, and *U*.

### 3.3. Efficiency Study of TPSLE Algorithm

In this section, an illustrative comparison among our proposed TPSLE algorithm, LHSD function in MATLAB, and SLE algorithm presented in Zhu et al. [20] is provided to show the significant savings achieved by our method.

The time consumptions of sampling designs using different algorithms are compared in Table 4. The computational time of them is measured on a PC with an Intel Core i3 3.3 GHz CPU. For sampling designs with various sizes accept *n* ≥ 10, the time of TPSLE is close to zero, which is more efficient than algorithm SLE especially for larger sampling size. For the sampling designs with size 32 × 6 and 256 × 8, TPSLE is even more effective than LHSD. When sampling dimension *n* ≥ 10, the computational time increases rapidly but is still acceptable.

## 4. Application Study of TPSLE Algorithm

In this section, five mathematical examples listed in Appendix A and one engineering problem are used to study the validity of TPSLE algorithm. TPSLE algorithm is applied to construct metamodels and deal with engineering optimization design problem in this work.

### 4.1. Comparative Study Based on Metamodel Accuracy

Sampling designs are very important for constructing metamodels. Poor sampling designs not only lead to poor accuracy of metamodels, but also reduce the efficiency. In this paper, five widely accepted mathematical examples are employed to test the accuracy of metamodels that are built with different sampling methods, that is, LHSD and TPSLE. As one of the most effective approximation methods, radial basis functions' (RBF) [23–25] interpolation is a better choice for constructing metamodels or finding the global optima of computationally expensive functions by using a limited number of sampling points. In this paper, RBF is used to construct metamodels and the basis function multiquadric is applied.

To make a fair comparison of two methods, the total number of sampling points (*m*_*p*_ = 64) is the same for each method in each tested problem. As mentioned in the last section, 50 times procedures are conducted for each sampling design and therefore there are 50 sets of accuracy results for each sampling method. The accuracy measures, NRMSE and NMAX [26, 27] (see Appendix B for definition) summarized in Table 5, are average values. Note that a value of zero for both accuracy measures, NRMSE and NMAX, would indicate a perfect fit.

From the results shown in Table 5, it is found that values of both NRMSE and NMAX by TPSLE algorithm are smaller comparable to those of LHSD function. It indicates that the metamodels based on TPSLE sampling algorithm can obtain better approximations for black-box functions. Such improved accuracy in metamodeling is attributed to better space-filling and LHD projective properties achieved by the TPSLE method. Therefore, the performance of TPSLE is better than LHSD function provided by MATLAB in constructing metamodels.

### 4.2. Engineering Problem

**Figure 10:**
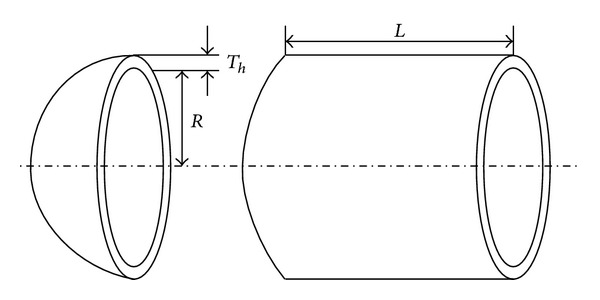
Diagram of pressure vessel design.

The performance of TPSLE algorithm is tested by a typical mechanical design optimization problem involving three design variables, that is, pressure vessel design. This problem is modified from the original problem recorded in [28–30]. The schematic of the pressure vessel is shown in Figure 10. In this case, a cylindrical pressure vessel with two hemispherical heads is designed for minimum fabrication cost. Three variables are identified: thickness of the head *T*_*h*_, inner radius of the pressure vessel *R*, and length of the vessel without heads *L*. In this case, the variable vectors are given (in inches) by

(9) $$\begin{array}{c} X=\left(T_{h},R,\text{L}\right)=\left(x_{1},x_{2},x_{3}\right). \end{array}$$

The objective function is the combined cost of materials, forming and welding of the pressure vessel. The mathematical model of the optimization problem is expressed as

(10) min⁡ f(X)=0.6224x1x2x3+1.7781×0.625x22+3.1661x12x3+19.84x12x2s.t. g1(X)=−x1+0.0193x2≤0,g2(X)=−0.625+0.000954x2≤0,g3(X)=1296000−πx22x3−43πx23≤0,g4(X)=−240+x4≤0.

The ranges of the design variables *x*_1_ ∈ [1.0,1.375], *x*_2_ ∈ [25,150], and *x*_3_ ∈ [25, 240] are used referring to the literature [30]. The minimum objective function value is 7021.3 declared in the literature [30].

The problem formulated above is a simple nonlinear constrained problem. Now assume the objective function defined by (10) is a computation-intensive function and thus the reduction of the number of function evaluations is considered. Hence, metamodel of objective function is constructed by RBF. Initial sampling points are generated by TPSLE algorithm. For comparison, sampling design method LHSD function is also employed. The average values of optimal results from 50 runs are listed in Table 6. It can be seen from the table that TPSLE outperforms LHSD in terms of both the minimum objective function value and the efficiency, that is, the number of design iterations. As shown in the table, the TPSLE method requires 7.28 iterations to reach the optimum, whereas the LHSD method needs 11.8 iterations. Therefore, the performance of TPSLE is better than LHSD function in solving engineering design optimization problem.

## 5. Conclusion

In this paper, a methodology for creating novel Latin hypercube designs via translational propagation algorithm (TPSLE) is proposed. The approach is inspired by the idea that a simple initial block with a few points generated by a novel algorithm SLE can be used as a building block to construct a near optimal Latin hypercube design. TPSLE algorithm offers a balanced trade-off between the efficiency and performance, that is, space-filling and the projective properties. The greatest advantage of the proposed methodology is that it requires virtually no computational time. In fact, no global objective functions are employed to optimize in TPSLE algorithm which is quite different from the existing LHD sampling methods. The performance of the sampling points generated by TPSLE is studied through comparison with other sampling methods under different test criteria, and the efficiency of TPSLE and other sampling methods is compared.

It is found that the space-filling and projective properties of sampling points using TPSLE are better than LHSD in most cases. In addition, though TPSLE algorithm is not as good as SLE in terms of performance of sampling points, the efficiency of TPSLE is further superior to SLE. TPSLE is a novel LHD sampling algorithm with acceptable space-filling and projective properties, and the efficiency of sampling algorithm TPSLE is superior. For the sake of examining the validity of the proposed TPSLE sampling algorithm further, five typical mathematical examples and one mechanical design optimization problem have been tested. The assessment measures for accuracy of metamodels, that is, NRMSE and NMAX, are employed. In contrast to the traditional sampling methods LHSD, TPSLE results in more accurate metamodels. Furthermore, TPSLE is superior in solving engineering design optimization problem on exploring global minimum of metamodels.

The proposed sampling algorithm TPSLE is a wise trade-off between performance and efficiency of sampling design and significantly outperforms the conventional sampling methods. However, there are still some shortcomings in TPSLE algorithm. Firstly, the performance of sampling points in high dimension is not good sometimes. Secondly, the sampling algorithm TPSLE cannot construct sampling points with arbitrary size directly. The problems mentioned above need to be resolved in future work.

## Figures and Tables

**Table 1:** Comparison of test criteria among TPSLE, SLE, and LHSD in two-dimension.

*n* = 2		TPSLE			LHSD			SLE		
*m*	Criteria	Best	Worst	Mean	Best	Worst	Mean	Best	Worst	Mean
16	*d*_min⁡_	0.177	0.140	0.146	0.178	0.061	0.114	0.198	0.140	***0.191***
	*CL*_2_	0.059	0.064	0.060	0.045	0.087	***0.054***	0.058	0.062	0.060
	*ϕ*_*p*_	5.816	7.255	7.025	5.062	8.074	6.790	5.060	7.155	***5.357***
	*U*	739.75	802.66	749.71	740.87	1398.3	951.87	708.19	754.56	***729.42***

32	*d*_min⁡_	0.113	0.070	0.087	0.095	0.033	0.057	0.156	0.088	***0.129***
	*CL*_2_	0.031	0.034	***0.032***	0.025	0.063	0.035	0.030	0.034	***0.032***
	*ϕ*_*p*_	8.999	14.713	12.392	10.558	29.996	18.045	6.688	11.314	***7.995***
	*U*	3957.9	4522.6	4216.8	4465.1	8371.5	5599.4	3820.0	3966.9	***3867.7***

64	*d*_min⁡_	0.099	0.035	0.064	0.045	0.014	0.028	0.105	0.035	***0.093***
	*CL*_2_	0.016	0.017	***0.016***	0.015	0.045	0.023	0.016	0.018	0.017
	*ϕ*_*p*_	10.467	29.021	18.075	20.458	32.345	26.618	9.732	28.622	***11.392***
	*U*	19278	21035	20007	24060	44498	30041	19179	19994	***19320***

128	*d*_min⁡_	0.052	0.017	0.036	0.024	0.008	0.014	0.074	0.025	***0.061***
	*CL*_2_	0.008	0.009	***0.009***	0.009	0.028	0.015	0.009	0.011	***0.009***
	*ϕ*_*p*_	19.779	58.042	31.482	42.235	129.40	72.667	13.739	40.477	***17.059***
	*U*	94780	100754	96565	119503	195253	152308	93131	94787	***93513***

**Table 2:** Comparison of test criteria among TPSLE, SLE, and LHSD in three-dimension.

*n* = 3		TPSLE			LHSD			SLE		
*m*	Criteria	Best	Worst	Mean	Best	Worst	Mean	Best	Worst	Mean
16	*d*_min⁡_	0.306	0.258	***0.281***	0.308	0.121	0.210	0.306	0.108	0.246
	*CL*_2_	0.097	0.115	0.103	0.074	0.132	***0.089***	0.087	0.095	0.092
	*ϕ*_*p*_	3.358	3.935	***3.619***	3.323	8.267	4.875	3.284	9.238	4.247
	*U*	339.41	382.95	***355.68***	344.65	557.14	411.31	357.67	438.01	376.71

32	*d*_min⁡_	0.221	0.153	***0.183***	0.184	0.084	0.124	0.261	0.077	0.182
	*CL*_2_	0.051	0.068	0.056	0.045	0.098	0.058	0.050	0.057	***0.053***
	*ϕ*_*p*_	4.653	6.716	***5.710***	5.529	11.926	8.268	3.847	13.064	5.988
	*U*	1645.2	1769.0	***1709.9***	1798.5	2689.1	2038.5	1673.7	1882.3	1743.5

64	*d*_min⁡_	0.147	0.077	0.110	0.106	0.047	0.075	0.206	0.038	***0.143***
	*CL*_2_	0.029	0.034	***0.032***	0.029	0.058	0.040	0.031	0.035	0.033
	*ϕ*_*p*_	7.016	13.431	9.704	9.455	21.358	13.639	4.977	26.128	***7.713***
	*U*	7642.1	8803.8	8148.1	8440.2	10480	9286.4	7617.8	8342.6	***7744.7***

128	*d*_min⁡_	0.073	0.038	0.058	0.067	0.027	0.047	0.149	0.014	***0.094***
	*CL*_2_	0.018	0.021	***0.019***	0.020	0.048	0.028	0.019	0.023	0.021
	*ϕ*_*p*_	14.028	26.862	18.825	15.130	37.412	22.068	6.744	73.901	***13.781***
	*U*	34282	38292	35798	37069	44138	40207	33268	39353	***33797***

**Table 3:** Comparison of test criteria between TPSLE and LHSD in high dimension.

Criteria method	Sampling size *m* × *n*										
	16 × 4	32 × 4	64 × 4	64 × 6	128 × 6	256 × 6	256 × 8	512 × 8	1024 × 10	1024 × 15	1024 × 20
*d*_min⁡_											
TPSLE	***0.395***	***0.346***	***0.230***	***0.433***	***0.429***	***0.294***	***0.468***	***0.476***	***0.500***	***0.538***	***0.678***
LHSD	0.308	0.205	0.138	0.274	0.210	0.163	0.273	0.225	0.273	0.518	0.635
*ϕ*_*p*_											
TPSLE	***2.601***	***2.963***	***4.575***	***2.441***	***2.444***	***3.653***	***2.334***	***2.354***	***2.349***	***1.825***	***1.118***
LHSD	3.301	4.971	7.394	3.702	4.811	6.221	3.696	4.486	3.700	1.956	1.380
*U*											
TPSLE	272.1	***1065.9***	***4634.5***	3049.4	***11108***	***44410***	35147	127547	427341	235684	187425
LHSD	***253.9***	1179.8	5145.5	***2701.6***	11116	45053	***30454***	***122542***	***372095***	***232627***	***169532***

**Table 4:** Comparison of time(s) among TPSLE, SLE, and LHSD.

Time(s) method	Sampling size											
	16 × 2	64 × 2	128 × 2	16 × 3	64 × 3	128 × 3	64 × 4	32 × 6	256 × 8	1024 × 10	1024 × 15	1024 × 20
TPSLE	0.0042	0.0100	0.0266	0.0039	0.0098	0.0579	0.0061	0.0050	0.0044	12.34	19.85	279.24
SLE	0.0058	0.0902	0.3802	0.0531	3.9341	35.948	1023.8					
LHSD	0.0040	0.0043	0.0048	0.0040	0.0054	0.0055	0.0052	0.0058	0.0080	12.48	12.77	12.83

**Table 5:** Comparison of metamodels accuracy between TPSLE and LHSD.

Function	Number of variables	NRMSE		NMAX	
		TPSLE	LHSD	TPSLE	LHSD
BR	2	0.0519	0.0973	0.0519	0.0973
AF	2	0.274	0.4012	0.274	0.4012
PEAKS	2	0.1153	0.1919	0.1153	0.1919
HN	3	0.3929	0.5755	0.3929	0.5755
MATH [22]	5	0.0383	0.0417	0.0383	0.0417

**Table 6:** Optimal results of pressure vessel design.

Method	Number of function evaluations	Number of design iterations	Variable design	Objective value
LHSD	16	11.8	[1.00, 44.50, 149.02]	7682.7
TPSLE	16	7.28	[1.03, 53.57, 72.34]	7044.0

## Appendices

### A. Mathematical Examples

Branin function (BR), *n* = 2:

(A.1) f(x)=(x2−5.14π2x12+5πx1−6)2 +10(1−18π)cos⁡(x1)+10,x1∈[−5,10], x2∈[0,15].

Alpine function (AF), *n* = 2:

(A.2) f(x)=sin(x1)sin(x2)x1x2,x1∈[0,10], x2∈[0,10].

Peaks function, *n* = 2:

(A.3) f(x)=3(1−x1)2·e−x12−(x2+1)2 −10(x15−x13−x25)·e−x12−x22 −13e−(x1+1)2−x22,x1∈[−3,3], x2∈[−3,3].

Hartman function (HN), *n* = 3:

(A.4) f(x)=−∑i=14ciexp⁡[−∑j=1nαij(xj−pij)2],x1∈[0,1], x2∈[0,1], x3∈[0,1],

where

(A.5) $$\begin{array}{c} \alpha_{ij}=\left[\begin{array}{ccc} 3 & 10 & 30\\ 0.1 & 10 & 35\\ 3 & 10 & 30\\ 0.1 & 10 & 35 \end{array}\right],\;\;p_{ij}=\left[\begin{array}{ccc} .3689 & .1170 & .2673\\ .4699 & .4387 & .7470\\ .1090 & .8732 & .5547\\ .03815 & .5743 & .8828 \end{array}\right],\\ c_{i}=\left[\begin{array}{c} 1\\ 1.2\\ 3\\ 3.2 \end{array}\right]. \end{array}$$

MATH function, *n* = 5:

(A.6) f(x)=∑i=15[310+sin(1615xi−1)+sin2(1615xi−1)],xi∈[−1,1], i=1,2,3,4,5.

### B. Metamodel Accuracy Measures

Root mean squared error (RMSE):

(B.1) $$\begin{array}{c} \text{RMSE}=\sqrt{\frac{\sum_{k=1}^{K}{\left[f\left(x_{k}\right)-\hat{f}\left(x_{k}\right)\right]}^{2}}{K}}, \end{array}$$

where *K* is the number of additional testing points and *f*(*x*_*k*_) and $\hat{f}\left(x_{k}\right)$ are the true function value and predicted metamodel value at the *k*th testing point *x*_*k*_, respectively.

Normalized root mean squared error (NRMSE):

(B.2) $$\begin{array}{c} \text{NRMSE}=\sqrt{\frac{\sum_{k=1}^{K}{\left[f\left(x_{k}\right)-\hat{f}\left(x_{k}\right)\right]}^{2}}{\sum_{k=1}^{K}{\left[f\left(x_{k}\right)\right]}^{2}}}. \end{array}$$

RMSE only calculates the error of functions themselves. However, NRMSE allows comparison of the metamodel error values with regard to different functions.

Maximum absolute error:

(B.3) $$\begin{array}{c} \text{MAX}=\max\left|f\left(x_{k}\right)-\hat{f}\left(x_{k}\right)\right|. \end{array}$$

Normalized maximum absolute error:

(B.4) $$\begin{array}{c} \text{NMAX}=\frac{\max\left|f\left(x_{k}\right)-\hat{f}\left(x_{k}\right)\right|}{\sqrt{\left(1/K\right)\sum_{k=1}^{K}{\left[f\left(x_{k}\right)-\overset{-}{f}\left(x_{k}\right)\right]}^{2}}}, \end{array}$$

where $\overset{-}{f}\left(x_{k}\right)$ is the mean of the actual function values at the *K* test points.

## References

[1] Gu L A comparison of polynomial based regression models in vehicle safety analysis.

[2] Koch PN, Simpson TW, Allen JK, Mistree F (1999). Statistical approximations for multidisciplinary design optimization: the problem of size. *Journal of Aircraft*.

[3] Mitchell TJ (1974). An algorithm for the construction of “D-optimal” experimental designs. *Journal of Technometrics*.

[4] Myers RH, Montgomery DC (1995). *Response Surface Methodology: Process and Product Optimization Using Designed Experiments*.

[5] Chen W (1995). *A robust concept exploration method for configuring complex systems [Ph.D. thesis]*.

[6] Johnson ME, Moore LM, Ylvisaker D (1990). Minimax and maximin distance designs. *Journal of Statistical Planning and Inference*.

[7] Morris MD, Mitchell TJ (1995). Exploratory designs for computational experiments. *Journal of Statistical Planning and Inference*.

[8] Simpson TW, Peplinski JD, Koch PN, Allen JK (2001). Metamodels for computer-based engineering design: survey and recommendations. *Engineering with Computers*.

[9] Rennen G, Husslage B, Van Dam ER, Den Hertog D (2010). Nested maximin Latin hypercube designs. *Structural and Multidisciplinary Optimization*.

[10] Rennen G, van Dam ER, Den Hertog D (2006). *Space-Filling Latin Hypercube Designs for Computer Experiments*.

[11] Koehler JR, Owen AB (1996). Computer experiments. *Handbook of Statistics*.

[12] Ye KQ, Li W, Sudjianto A (2000). Algorithmic construction of optimal symmetric Latin hypercube designs. *Journal of Statistical Planning and Inference*.

[13] Jin R, Chen W, Sudjianto A (2005). An efficient algorithm for constructing optimal design of computer experiments. *Journal of Statistical Planning and Inference*.

[14] Bates S J, Sienz J, Toropov V V (2004). Formulation of the optimal Latin hypercube design of experiments using a permutation genetic algorithm. *AIAA Journal*.

[15] Liefvendahl M, Stocki R (2006). A study on algorithms for optimization of Latin hypercubes. *Journal of Statistical Planning and Inference*.

[16] Grosso A, Jamali ARMJU, Locatelli M (2009). Finding maximin latin hypercube designs by Iterated Local Search heuristics. *European Journal of Operational Research*.

[17] Jourdan A, Franco J (2010). Optimal Latin hypercube designs for the Kullback-LEIbler criterion. *AStA. Advances in Statistical Analysis.*.

[18] van Dam ER, Husslage B, den Hertog D (2007). Maximin Latin hypercube designs in two dimensions. *Journal of Operations Research*.

[19] Viana FAC, Venter G, Balabanov V (2010). An algorithm for fast optimal Latin hypercube design of experiments. *International Journal for Numerical Methods in Engineering*.

[20] Zhu H, Liu L, Long T, Peng L (2012). A novel algorithm of maximin Latin hypercube design using successive local enumeration. *Engineering Optimization*.

[21] Hickernell FJ (1998). A generalized discrepancy and quadrature error bound. *Mathematics of Computation*.

[22] Mullur AA, Messac A (2005). Extended radial basis functions: more flexible and effective metamodeling. *AIAA Journal*.

[23] Babu GS, Suresh S (2013). Sequential projection-based metacognitive learning in a radial basis function network for classification problems. *IEEE Transactions on Neural Networks and Learning Systems*.

[24] Yao W, Chen XQ, Huang YY, van Tooren M (2014). A surrogate-based optimization method with RBF neural network enhanced by linear interpolation and hybrid infill strategy. *Optimization Methods & Software*.

[25] Vuković N, Miljković Z (2013). A growing and pruning sequential learning algorithm of hyper basis function neural network for function approximation. *Neural Networks*.

[26] Jin R, Du X, Chen W (2003). The use of metamodeling techniques for optimization under uncertainty. *Structural and Multidisciplinary Optimization*.

[27] Mullur AA, Messac A (2006). Metamodeling using extended radial basis functions: a comparative approach. *Engineering with Computers*.

[28] Coelho LDS (2010). Gaussian quantum-behaved particle swarm optimization approaches for constrained engineering design problems. *Expert Systems with Applications*.

[29] Cao YJ, Wu QH Mechanical design optimization by mixed-variable evolutionary programming.

[30] Wei X, Wu Y, Chen L (2013). A global optimization algorithm based on incremental metamodel method. *China Mechanical Engineering*.

